# Extra-Low-Frequency Pulse Stimulated Conformational Change in Blood-Cell Proteins and Consequent Immune Activity Transformation

**DOI:** 10.1109/JTEHM.2020.2963894

**Published:** 2020-01-09

**Authors:** Ibtisam A. Abbas Al-Darkazly, S. M. Rezaul Hasan

**Affiliations:** Center for Research in Analog and VLSI Microsystems Design (CRAVE), School of Advanced TechnologyMassey University 6420 Auckland0632New Zealand

**Keywords:** Electrical stimulation, buffy coat, amino acid (AA), co-receptor CCR5, CD4 glycoprotein, nuclear pore complex (NPC), nucleoporin Nup153, immuno-fluorescence, antibody (Ab)

## Abstract

*Objective:* investigation of the extra-low-frequency (ELF) stimulation effect on blood-cell proteins, that causes variation in its electrostatic-state. A hypothesis that this results in the conformational change in the blood-cell proteins which could enhance immune activity is explored. Since HIV-1 and host-cell engage through charge-charge interactions, an electrical-pulse may cause charge redistribution, hypothetically resulting in host-cell proteins to be isolated from viral access. *Methods:* Buffy coat samples were exposed to ELF square waveform pulses of 5Hz, 10Hz and 1MHz, for 2-hours, and were then examined using immunofluorescence technique. The expression of glycoprotein CD4, and co-receptor protein CCR5, were investigated. Also, the binding activity of the N-terminal domain of CCR5 and the distribution of the nuclear-pore-complex (NPC) transport factor, FGNup153 were investigated. Comparison with control samples were carried out. *Results:* Increased CD4 count, which could enhance the immune system. In addition, the inability of N-terminus-specific antibody *3A9* to bind to CCR5 N-terminal, could be due to the interactions with the ELF electric-field, which may also hypothetically inhibit HIV-1 attachment. Furthermore, the electrostatic interactions between the ELF pulse and the FGNup153 induces redistribution in its disorder sequence and possibly causes conformational change. This could possibly prevent large virus particle transport through the NPC. Conclusion: Novel concept of ELF stimulation of blood cellular proteins has been developed leading to transformation of immune activity. Clinical-Impact: The translational aspect is the use of ELF as an avenue of electro-medicine and the results are a possible foundation for the clinical application of ELF stimulation in immune response.

## Graphical abstract

In this work, the underlying domain region protein-protein electrostatic interaction mechanisms of the HIV-1 virus and host cells is explored. A novel concept of low-voltage ELF stimulation of human blood cellular proteins was developed to investigate the hypothesis of conformational change owing to the conductivity and permittivity properties of these proteins, leading to transformation of immune activity. The results motivate possible clinical application of ELF electrical stimulation for enhancing immune activity (e.g., through increased cell surface CD4 expression) or possibly inhibiting virus and host-cell interaction, which could be employed in-vivo as an anti-HIV1 treatment.

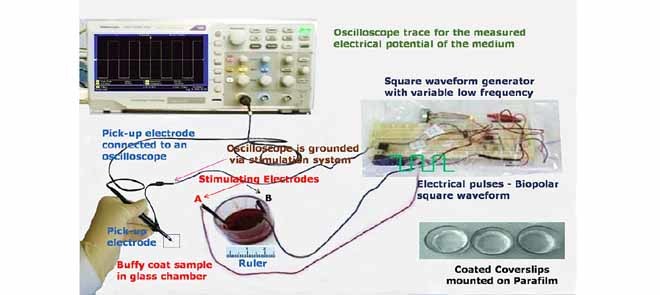

## I. Introduction

The electro-active biological cells inspire researchers to investigate its electronic characteristics for translational aspects in health and medicine. The application of electrical force to stimulate cellular reactions and activate bio-cells to recover from a disorder or disease is one such consideration. There is potential for treating a variety of chronic diseases especially when therapeutic drugs become ineffective, such as the case of drug resistant HIV-1 viral disease. Electrical stimulation for disease treatment, has long been known to have an important effect on living creatures. It can influence the cell function by inducing various types of responses which may enhance [1] or inhibit [2] various molecular mechanisms. This depends on the type and intensity of the electro-force, frequency, wave-shape, duration/duty-cycle and amplitude, which induce electronic-transfer interactions. External electrical force alters cellular membrane potential resulting in enhanced cellular functionality [3], and consequent conformational changes of pore proteins, enzymes, phase transitions of membranes, and ionic pumping across the cell membrane with structural rearrangements. These interactions involve frequency dependent polarization due to the conductivity and permittivity of biological cells, tissues, blood and proteins [4], [5]. The effect on infected lymphocytes, comprising complex interactions, depends on the cells’ functional state. Several studies have demonstrated that electro-forces can influence the immune responses of animals and humans by inducing various immune response elements that enhance the production of antiviral substances (molecular species) [2], [6]. Proteins are involved in cellular interaction with different signals, which initiate events from the extracellular to the intracellular. These signals trigger most cellular processes and can induce conformational (structural) change in the cellular proteins which stimulates reactions that affect the functions of many other proteins that are involved in numerous diseases [7]. With respect to signal transfer, CD4 receptor, co-receptors CCR5 and nuclear pore complex (NPC) component proteins of the lymphatic system, are essential targets for HIV-1 infection. HIV-1 infection and replication is characterized by directly infected CD4+ T cells while utilizing cell surface co-receptor CCR5 for specific binding process and entry. This involves protein-protein interaction predominantly by charge-charge electrostatic interaction and conformational (structural) changes [8], [9] which play a role in the binding process, sustaining interactions with the target cells, and mediating the HIV life cycle replication. The virus then traffic from the cellular periphery to the desired regions of host DNA within the nucleus [10], utilizing its nuclear transport factor nucleoporin153 (Nup153) proteins causing progressive reduction of the CD4+ T cells which result in AIDS. HIV-1 infection therefore, is a cytopathic viral infection and its study is focused on exploring the molecular biology of the virus within the host cell that can be used for HIV-1 therapy. Consequently, research has focused on immune-based therapies, antibodies that target host proteins, such as the use of combinations of co-receptor binding inhibitors for most active anti-HIV-1 therapy; these inhibitors become the drugs of choice in pharmacological approaches for HIV-1 patients [11]. In this work the effect of low frequency electrical force on cell surface expression of CD4 and CCR5, protein–protein interfaces within the domain regions of CCR5 proteins and Nup153, one of the NPC components, is investigated in-vitro. This process, that involves alignment and polarization of polar and charged protein molecules may stimulate immune response, and could be a mechanism prompting conformational changes that could disturb the HIV-1-host-cell interaction for a period of time. The experimental procedures carried out in this work are thus geared towards verification of protein transformations of the domain region host cellular proteins that could inhibit molecular level viral infection mechanisms.

## II. Predominant Regions of CCR5 and Nup153

### A. Chemokine Receptor CCR5

**FIGURE 1.**
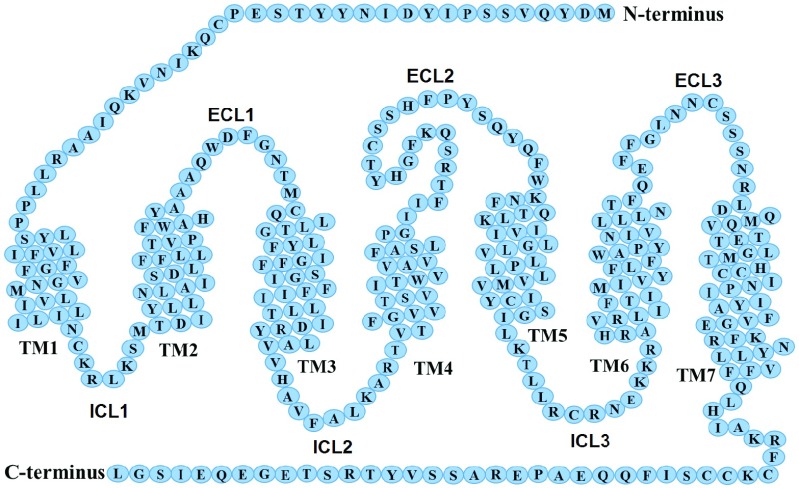
Co-receptor CCR5 structure and its sequence. The image outlines the residues of N-terminus, C-terminus, the 7-TM regions, the extracellular loop (ECL), and the intracellular loop (ICL). The featured model is a sketch based on the CCR5 model from [13].

Fig. 1 shows the protein structure of CCR5. These proteins consist of motifs of charged or hydrophobic regions, and are considered important for the functional response of the CCR5 and for the HIV-1 binding activity [12]. Direct effects on CCR5 proteins could be the major mechanism through which to prevent the virus from binding on the cell. Hence, CCR5 inhibitors based on conformational changes of the CCR5 are the target of anti-HIV-1 approaches. This approach alters the interaction with the HIV-1 envelope protein gp120 [13], and has been used to develop pharmacological agents able to inhibit viral infection in the early stages [14]. Several studies have demonstrated that CCR5 N-terminal regions play a key role in the binding activities within the third variable region (V3-loop) of gp120, allowing the virus to use CCR5 [9]. Specific amino-acids within the CCR5 N-terminal, comprising negatively charged and tyrosine residues, are vital for CCR5-mediated entry of CCR5 specific and CCR5/CXCR4 specific HIV-1 strains. The authors in [15] have shown that the overall positive charge of the V3-loop is interconnected with the negatively charged region of CXCR4 or CCR5. Earlier, studies in [16], showed that the N-terminal site of CCR5 is rich in tyrosine (Y) and acidic amino acids mediating the link between gp120 and CCR5 which are essential in HIV-1 infection and chemokine binding. It was found that charge-charge interaction determines the interaction between the predominantly positive V3-loop and the predominantly negative N-terminal peptide of CCR5, and thus electrostatics drives the binding-site (epitope) recognition and binding activities. The authors in [17] reported that monoclonal antibody (*mAb*) *3A9* that recognize the conformational epitopes on CCR5 at the N-terminus, can block HIV-1 entry, and that, mutation of the N-terminal motif Y^10^D^11^ prevented HIV-1 entry into transfected cells with CCR5 specific and CCRR5/CXCR4 specific HIV-1 strains. They demonstrated that the conformational contact sites of CCR5 with the *mAb 3A9* represent sites on CCR5 that are essential for HIV-1 entry. In pharmacological approaches, additions or substitutions of a few charged amino-acid residues at the termini of extracellular terminal domains are used to alter the net charge and induce conformational change that blocks the HIV-1 interaction with a host-cell receptor, which could prevent HIV-1 viral entry. It is thus clearly evident that the interactions between the CCR5 N-terminal and the viral V3-loop, is predominantly by protein charge-charge electrostatic interactions. Interestingly, since this binding process is dominated by charge-charge electrostatic interaction, redistribution of the charge in the CCR5 N-terminal domain in response to an external/applied ELF electric field may result in conformational (structural) changes of its proteins. This could hypothetically disrupt the interaction between HIV-1 virus and host-cell, and hence, inhibit HIV-1 infections and ligand binding affinity. In this work, alterations of the predominantly polar and charged amino-acids of CCR5 N-terminal domains in response to ELF electric fields, is thus used, to investigate the CCR5 N-terminal binding activities utilizing antibody *3A9* that recognizes epitopes in the N-terminal domain of CCR5.

### B. The NPC Features and its Important Nup153 Factor

**FIGURE 2.**
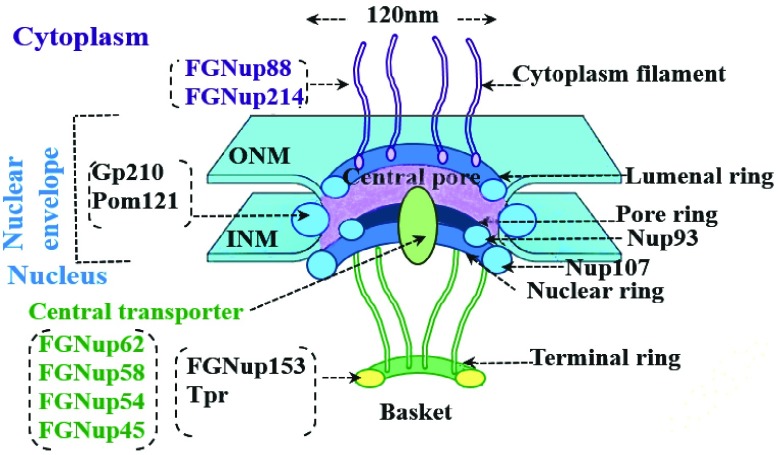
The different layers in the NPC indicating the various FGNups. ONM and INM are the outer and inner membranes respectively. The featured model is a sketch based on the model of the NPC from [18].

**FIGURE 3.**
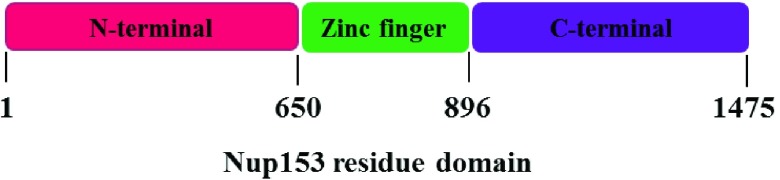
The tripartite structure of FG-Nup153, comprising N-terminal domain, zinc finger domain, and C-terminal domain, based on NPC model.

The HIV-1 replication involves the translocation of viral particle into the host nucleus for expression of its genome. The HIV-1 life-cycle is thus accomplished by utilizing host nuclear transport mechanism to enable the passage of its large molecules, particle pre-integration complex (PIC) and the viral RNA through nuclear pores. The nuclear pores comprises of a macromolecular assembly, the nuclear pore complex (NPC). Active translocation requires binding to specific nuclear transport components, to overcome the permeability barrier of the NPC. It is facilitated by Importins and Exportins [19] that attach to certain signals and carry them through the NPCs. This process is completed through a sequence of interactions with various NPC proteins, called nucleoporin (Nup) [20]. Around 30% of Nup proteins are rich in phenylalanine-glycine (FG) repeating domains within their amino-acid (AA) sequence hence, termed FG-Nups. These FG-Nup proteins are dynamic components and highly flexible structures, characterized as intrinsically disordered proteins [21]. Fig. 2 shows the NPC structure. Its sequences comprise FG repeats, that are associated with amino-acid linkers, regulating the formation of the FG Nups network at the centre of the NPC. They act together with translocating particles to re-arrange the permeability barrier and organize the selective translocation through the NPC. Momentary electrostatic interactions between transporters (cargo complexes) and disordered domains of FG-Nups are considered the main driving force that stimulate active translocation of cargo through the NPC. The interaction of the FG-Nups with specific nuclear transport receptors (NTRs) mediates passage of NTRs cargo through the central channel of the NPCs. One of the highly dynamic components of the NPC is the FG-Nup153 that is localized specifically to the distal-ring of the NPC basket (Fig. 2). FG-Nup153, plays a role in the import and export of the infecting HIV-1viral particle into the host. It comprises three different domains as shown in Fig. 3.

**FIGURE 4.**
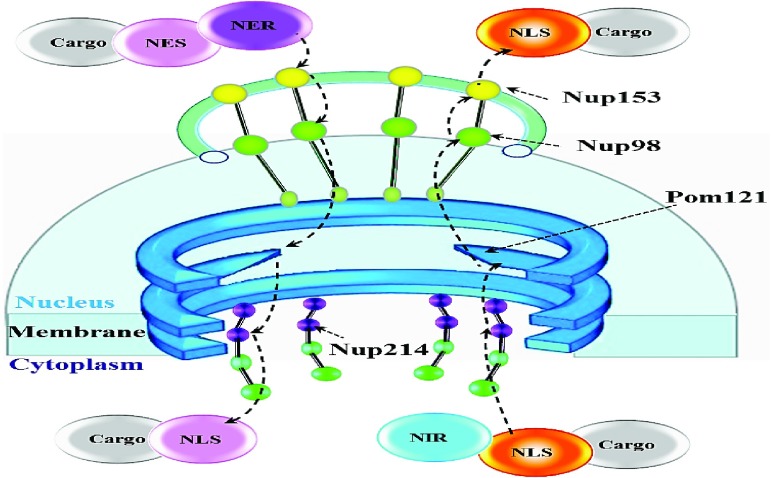
NPC model for translocation of large macromolecular proteins that are recognised by nuclear transport receptors (NTRs) and passes them through the central channel. FG-Nup153 are attached to the nuclear rim of the NPC and are vital in the import (orange) and export (pink) events of the cargo complex. NIR and NER are the Nuclear Kap/Importin and Kap/Exportin receptors respectively [25].

The nuclear import and export receptors interact with several regions of FG-Nup153. The HIV-1 virus is therefore incapable of passing the large infecting particle into the host independent of nuclear transport components, particularly FG-Nup153. The FG-Nup153 has potential role during HIV-1 PIC sub-viral particle nuclear import [22]. HIV-1 Vpr is able to bind to FG-Nup153 N-terminal domain [23], while the HIV-1 IN (integrase protein) particle binds directly with the C-terminal domain of FG-Nup153. Depletion of FG-Nup153 expression inhibits HIV-1 infection by inhibiting nuclear import. The HIV-1 virus also utilizes a cellular protein export pathway, to transport unspliced late viral mRNA [24] transcripts to the cytoplasm. The virus proteins are encoded by these large mRNA transcripts in the nucleus. The export of viral RNA is facilitated by the viral RNA-binding protein, Rev [26], coded in all unspliced viral transcripts. Rev shuttles between the nucleus and the cytoplasm prompting rapid nuclear export when binding to a target protein. The authors in [27] demonstrated that FGNup153 is actually involved in the mechanisms of protein and RNA in nuclear export, as well as in the HIV-1 Rev protein export pathway. This study reported that FG-Nup153 is also important in the nuclear import activities as shown in Fig. 4. Consequently it is possible to hypothesize that this transport mechanism could be disturbed through electrostatic interactions between external/applied electric field (using ELF electrical pulses) and the FGNup153 domains, to establish effective temporary permeability barrier across the NPCs. This could inhibit the reorganization of NPCs and hence, the import and export of high weight molecular HIV-1 particles.

## III. Concept Development for Electric Field and Frequency Dependent Polarization

Proteins are dynamic molecules with conductivity and permittivity properties [28]. This is due to the different charge distributions, involving polar and charged molecules in its structure which allow proteins to assemble with other proteins by electrostatic interaction undergoing structural rearrangements in response to intracellular and extracellular signals. The predominant charged groups carboxyl (COO^−^) and amino (NH3^+^) in the proteins’ structure possess dielectric properties due to the presence of dipole moment [4]. In addition, these ionisable groups, in solution, produce strong electrostatic interactions which possesses another dipole moment. The different compositions of the side chains also results in different charge distributions such as for a polar amino acid (e.g., Tyrosine Y, Glutamine Q, Serine S, Asparagine N, Threonine T, and Cysteine C), negatively charged proteins for acidic amino-acids (e.g., Aspartic D and Glutamic E) and positively charged proteins for basic amino-acid such as Lysine K [29], [30]. Consequently, in the presence of an external/applied ele**c**tric field, the randomly orientated polar molecules as well as the positively and negatively charged molecules within the protein rotate, move, align and polarize, and are thus reorganized, producing an internal dipole along-with internal electric field, which is opposite to the external electric field. This mechanism could inhibit the protein charge–charge electrostatic interaction of host-cell and the HIV-1 virus. The degree of polarization is dependent on the magnitude of the applied/external electric field and its oscillation frequency. When the applied electric field (E) is increased, the polarization P (P $= \varepsilon _{0}$ [$\varepsilon _{\mathrm {r}}-1$] E) of the proteins increases, where $\varepsilon _{0}$ is the permittivity of free space and $\varepsilon _{\mathrm {r}}$ is the relative permittivity of the material [31]. However, electrical breakdown occurs when the high electric field exceeds the dielectric strength of the protein material. This arises when a given voltage is applied for a long duration, that disturbs the orientation of the dipoles and the dielectric material is incapable of reaching a state of equilibrium [32]. Hence, there is a limit to the amplitude and duration of the electric-field for any desirable conformational change in proteins for immune activity when exposed to electrical force. Similarly, if the electrical force involves pulses of various frequencies, polarization will follow the oscillation characteristic of the electric field. At very low frequencies proteins exhibit a large permittivity, because the dipole present in proteins has enough time to align and polarize in response to the applied field, before its change in direction. At intermediate frequencies, the dipole movement decreases, and does not have enough time to follow the changing direction of the electric field, and, thus the polarization of the dipoles decreases. Proteins in this case have lower permittivity. At very high frequencies, the dipoles does not have enough time to align and are totally unable to respond to the applied electric-field and polarize, causing dielectric heating, and hence, dielectric loss takes place. This behaviour follows the Debye relaxation that is characterized by the relaxation time $\tau $, [4] and can be expressed by:

\begin{align*} {\varepsilon }'(\omega)=&\varepsilon _{\infty } +\frac {\left ({{\varepsilon _{S} -\varepsilon _{\infty }} }\right)}{1+\omega ^{2}\tau ^{2}} \tag{1}\\ {\varepsilon }''(\omega)=&\frac {\left ({{\varepsilon _{S} -\varepsilon _{\infty }} }\right)\omega \tau }{1+\omega ^{2}\tau ^{2}}\tag{2}\end{align*}

where, $\varepsilon '$ is the permittivity, $\varepsilon ''$ is the dielectric loss, $\varepsilon _{\mathrm {s}}$ is the low-frequency relative permittivity, $\varepsilon _{\infty }$ is the high-frequency relative permittivity and $\omega $ is the angular frequency. If the relaxation time is ≤ the period of electric field oscillations, there is minimum dielectric loss. Whereas, if the period of oscillating field is < the relaxation time, the dipole polarization can no longer follow the oscillating frequency, resulting in maximum dielectric loss. Schwarz showed that the dipole polarizability of the bio-molecule (protein) is frequency-dependent and can be given by,

\begin{equation*} \alpha =\frac {\alpha _{O}}{1+j\omega \tau }\tag{3}\end{equation*}

where, $\alpha $ is the dipole polarizability and $\alpha _{o}$ is the low frequency polarizability. The period of any applied electric field thus should be greater than the relaxation time of protein dipoles for desired structural change in the protein.

The conductivity and permittivity properties present in proteins, thus enables functional interface with other proteins to perform the required cell functions. The protein size may also effect its electrochemical properties and hence its interface with other proteins. The type, intensity and duration of any applied therapeutic electrical stimulation force, therefore, must be selected carefully to stimulate an appropriate curative action and must not be so strong that it would produce undesirable responses (*side-effects*). Considering proteins as dielectric materials, when low frequency pulses are applied, proteins usually present a large permittivity with little or no dielectric loss, while at high frequencies significant dielectric loss may take place, which can affect the dielectric properties of the proteins and hence the cell function. Also, applying a low intensity electric field can enhance the transport of ions across the cell membrane, while at high intensities an electric field can cause dielectric breakdown [4]. This is due to the fact that at a low intensity electric field, nano-scale pores form in the cell membrane temporarily, while at a high intensity electric field, the cell is unable to recover from the pore formation, causing possible cell death. Thus, high intensity electric-fields are used in electroporation therapies for treating a variety of cancerous pathologies [33]. The movement of charged molecules and free ions, in response to an applied external electric field can also be a useful mechanism to induce an action comparable to the normal cell endogenous electric field. The endogenous field, that induces a variation in the membrane potential due to the movement of charged residues in intrinsic plasma membrane proteins, is important in biological processes such as wound healing and tissue regeneration. An additional notable feature of applied electrical stimulation is its cellular specificity. The parameters of the generated electrical pulse to be applied can be designated and selected to stimulate only one particular tissue such as blood while leaving other organelle and molecular species unaffected [34]. In the electrical stimulation approach, the type of waveform must also be carefully considered. Periodic stimulation waveforms of certain frequencies, can interact with the intrinsic periodic oscillations of biological cell networks. This can enhance the intrinsic cellular oscillatory activity [35], a phenomenon which describes the natural frequency of the human body, that constitutes an essential process in the living organism for cell communication [36]. Since disease can disrupt the biochemical systems in cells, which inhibit the normal protein synthesis in lymphocytes, applying certain external stimulation frequencies can enhance the biochemical parameters of blood cells and normalize protein synthesis [37]. The mode and the duration of the electrical stimulation pulse are also important. Clinically, using long duration monopolar mode leads to charge accumulation at the electrode-skin site, causing muscle contractions which may damage the tissue, while with a long duration bipolar mode of operation, each pulse is followed by a pulse of reversed polarity which ensures charge balancing and hence, prevents damage that may occur at the electrode-skin interface [33].

Based on the above, theoretically, a low frequency square waveform may diminish the interaction between the domain regions of the virus and the host cells through electrostatic interactions causing protein confrmational (structural) change, so that, the HIV-1 virus cannot mutate and develop resistance in the absence of the relevent host-cell protein structure as in traditional pharmaceutical approaches. However, it is not expected to seriously disturb the bio-chemical protein structure since protein molecules have non-polar hydrophobic amino-acid residue which builds up their core, while the polar and charged amino-acid residues are mostly located on the surface of the molecule.

## IV. Materials and Methods for In-Vitro Biological Experiments

### A. Blood

Freshly sourced human leukocyte-rich buffy coat samples from a healthy volunteer was obtained through the New Zealand Blood bank Services (NZBS). The sample is supplemented with Anticoagulant Citrate-Phosphate-Dextrose (CPD) solution for preservation and to prevent clotting. The samples are employed for the ELF stimulation test on cell surface receptors CD4, co-receptors CCR5 and on the CCR5 N-terminal domain, as well as, the distribution of FG-Nup153.

### B. Immunological Reagents and Materials

The following fluorescent antibodies (*Ab*s) and materials were procured from Medi’Ray New Zealand Ltd: [*conjugated Ab FITC anti-human CCR5*] (Cat: 313705), [*conjugated Ab Alexa Fluor 488 anti-human CD4*] (Cat: 317419), purified primary antibody against CCR5 N-terminal, [*unconjugated Ab 3A9*] (Cat: 634501) and [*conjugated Ab Alexa Fluor 488 Goat anti-mouse IgG*] (Cat: 405319). Cell fixation buffer: 4% Paraformaldehyde (PFA) (Cat: 420801), plastic coverslips (No.: BAH446900000), glass slides (No.: S8400-1PAK), Poly-L-lysine coating solution (No.: P4707), and anti-fade mounting medium fluoro-shield (No.: F6937) were procured from Sigma-Aldrich. In addition, purified primary antibody against NPC proteins, [*unconjugated anti-Nup 153*] (Cat: 906201), [*conjugated Ab Alexa Fluor 555 Goat anti-mouse IgG*] (Cat: 405324) and $4^\prime ~6$-diamidino-2-phenylindole (DAPI) (Cat:422801) were procured from Medi’Ray, along with 3 glass chambers-3cm, forceps for mounting coverslips, and clear finger nail polish to seal coverslips on slide. Phosphate buffer saline (1X PBS) and Serums, HCl acid, Ethanol and Methanol were obtained through courtesy of the Massey Human Nutrition Lab, along with all other apparatus such as beakers, Parafilm, polypropylene tubes, 1 mL pipette, 100 $\mu \text{L}$ pipette, $20~\mu \text{L}$ pipette, $2~\mu \text{L}$ pipette and their pipette tips.

### C. The Electrical Test Chamber

**FIGURE 5.**
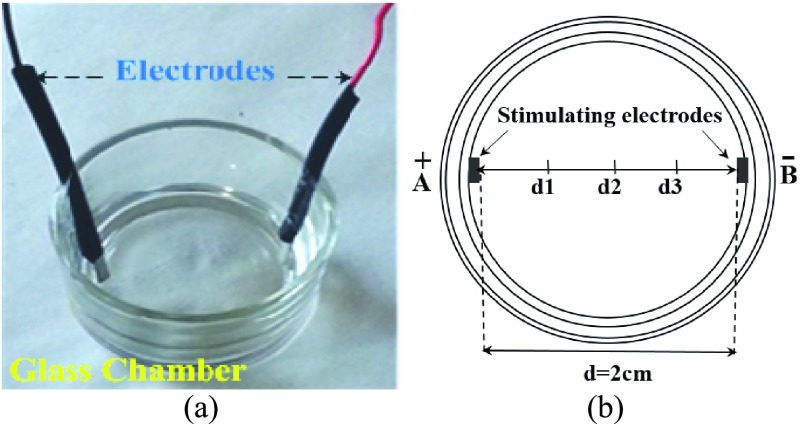
(a) The electrical test glass chamber with stainless steel wire electrodes. (b)Top-view drawing indicting pickup electrode probing locations along the horizontal axis where potential measurements were done in the medium.

The electrical test chamber was custom designed using a 30ml glass chamber 3cm in diameter, along-with two stainless steel wire electrodes and were autoclaved for the electrical test. Flat electrodes were employed in order to obtain an uniform electric field intensity over the blood sample. The electrode material was chosen considering *non-invasive in-vitro* application as well as electrical conductivity, corrosion resistance and cost. Fig. 5 shows the electrical test glass chamber with electrodes.

### D. Electrical Stimulation Procedure

**FIGURE 6.**
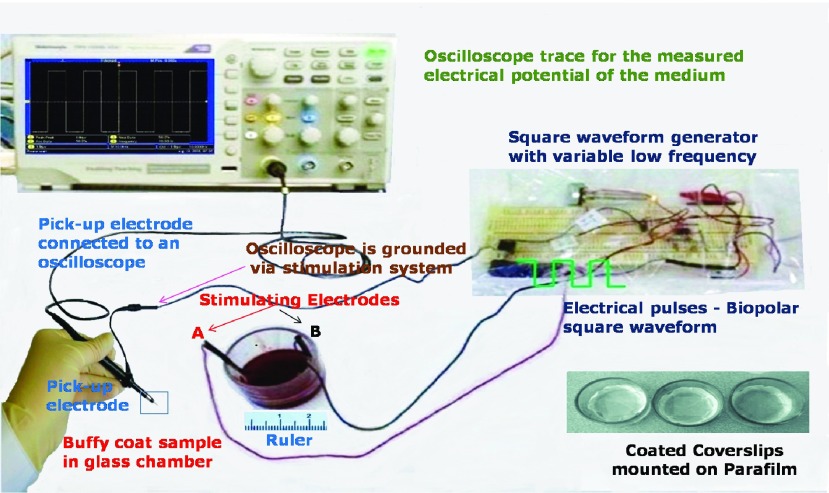
Experimental set-up for electrical stimulation test, and, measurement of electric-field intensity in the medium. A pickup electrode is connected to an oscilloscope to manually measure the electric potentials and field strengths in the medium at the defined points between the stimulating electrodes A and B.

The parameters for electric stimulation were chosen based on the electrical properties of proteins along with the electric field and polarization concepts introduced in section III. Buffy coat samples were exposed for 2h to 2Vpp low frequency bipolar square waveform pulses of 5Hz, 10Hz and 1MHz in the electrical test chamber. Unstimulated samples as controls were also prepared under rigorously same experimental conditions with chamber and electrodes but without being subjected to ELF waveforms, for comparison with stimulated samples. Considering cellular dimensions in the range of $8\mu \text{m}$ to $10\mu \text{m}$ and separation of electrodes in centimetres, a 2V signal will generate electric fields in the range of around 1V/cm. It is far less than high field intensities in the range of 300kV/cm that disturbs the protein’s dipole orientation and dielectric properties and causes cellular dielectric breakdown [4]. Hence the chosen pulse amplitude can be considered clinically safe. The protein polarization, P also depends on the electric field (E) as discussed in section III, and the strength of the applied electric field for a 2V stimulation signal can result in sufficient polarization of the protein molecule. The duration of cell exposure was decided so as to give sufficient time for species expression (e.g., CD4 and CCR5), as well as, for rotation and realignment of charged and polar molecules for observable experimental outcomes. Electrical pulses in the ELF range (5Hz and 10Hz) was mostly chosen considering the possibly large relaxation time of dipoles in proteins and the dipole polarizability based on (1) and (3) respectively in section III. A higher frequency (1MHz) was also employed to observe the difference in response experimentally. Electric-fields and current are produced through the two electrodes in direct contact with the blood samples. The pulses were applied using a variable frequency square waveform generator developed in the lab. The bipolar pulses ensures charge balancing as discussed in section III. For health and safety, all the experiments were performed under a laminar flow hood at 25°C (room temperature). Fig. 6 shows the experimental set-up for the electrical stimulation treatment.

### E. Immunofluorescence Microscopy Assay Technique

**FIGURE 7.**
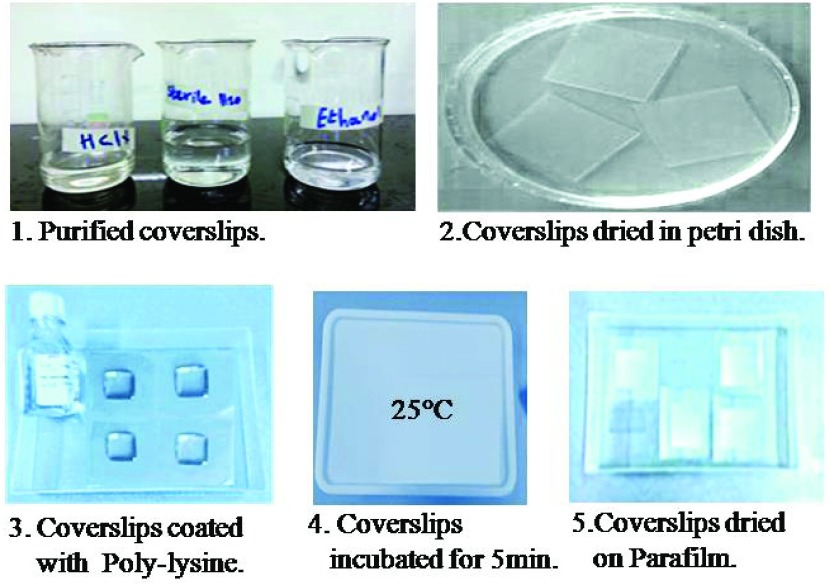
Purifying and coating coverslips with HCl and poly-lysine respectively, to assure cell adhesion.

**FIGURE 8.**
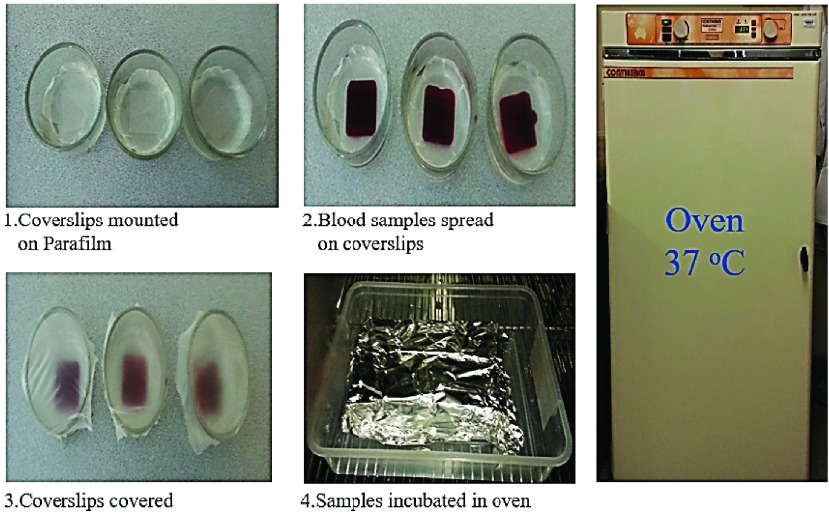
Cell adhesion on coverslips. Buffy coat samples for electrical stimulation and also as control (for the unstimulated cells case) were spread and plated on coated coverslips, and then incubated in oven for 30 min. at 37 °C.

Immunofluorescence analysis was performed for electrically stimulated cells and for the unstimulated cells as controls according to the manufacturer (BioLegend) protocol [38], under rigorously same conditions and was modified for achieving optimum staining results. All the apparatus were autoclaved for the biological tests. It is necessary to purify the coverslips before cell adhesion, and hence, the coverslips were immersed in diluted solution of 10% hydrochloric acid (HCl) for 2h, rinsed 3 times for 15 min. with distilled water and stored in 70% ethanol solution. Coverslips were then removed from ethanol and dried in a petri-dish at room temperature as indicated in Fig. 7 (steps 1–2). Guidance for purifying the coverslips (protocol 5) provided in [39], was followed with a slight modification. In addition, the coverslips were coated with poly-lysine to promote cell attachment and adhesion. A simple technique was employed to prevent the coverslips from moving and also to control the amount of poly-lysine solution, by mounting the coverslips on Parafilm. After mounting purified coverslips (22mm$\times 22$mm$\times0.157$mm), around $200\mu \text{l}$ poly-lysine was added to just cover the surface of the coverslips and were subsequently incubated for 5min. at room temperature, in accordance with the supplier (Sigma) protocols. The solutions were then aspirated off using sterilized micropipette, and the coverslips were dried for 2h at room temperature before use as indicated in Fig. 7 (steps 3–5). Initially, buffy coat samples for electrical stimulation and also as control (for the unstimulated cells case) were spread and plated on the coated coverslips in the glass chamber and then covered by aluminum foil and incubated (in Contherm Thermotec 2000 oven) for 30 min. at 37 °C as shown in Fig. 8 (steps 1–4) under rigorously same conditions. It is estimated that roughly $1 \times 10^{5}$ cells were placed on each coverslip following BioLegend [38] protocol in order to achieve optimum cell density. After incubation, the coverslips were then rinsed briefly in 1X PBS using a sterilized micropipette. In this part of experiments, to confirm the influence of the electrical stimulation on the cell samples, cells fixation was employed for the stimulated cells as well as for the unstimulated cells as controls. The cells fixation may be done by either using PFA (of varying concentration) in PBS or cold 100% methanol, depending on the target protein and antibodies and in accordance with the supplier, BioLegend [38] protocol. If the target is the cells’ intracellular protein, such as protein inside NPC, cells are fixed with 4% PFA in PBS, along-with cell permeabilization using 0.5% Triton X-100 in PBS. Methanol fixed samples do not require permeabilization [40]. On the other hand, for the cell surface expression of protein, as in the case of CD4 and CCR5, permeabilization is not required and cell fixation may be performed using 1% PFA in PBS, which is again based on the BioLegend Immunofluorescence microscopy protocol [38]. Hence, For the analysis of cell surface expression intensity and distribution of CD4 or CCR5, unstimulated (virgin) cells and electrically stimulated cells were fixed by incubation for 15 min. at room temperature with 1% PFA and were rinsed 3 times for 5 min each with 1X PBS. Cells were then blocked in 5% FBS in PBS, at room temperature, for 1h in order to prevent unspecific binding of the antibodies. Thereafter, the samples are labelled with the specified antibodies. For optimum results, all antibodies were diluted and titrated to the recommended concentration/dilution in 1X PBS in 5% BSA/FBS before use. $200\mu \text{l}$ of diluted antibody were then added to each coverslip mounted on Parafilm. Only the coverslips with uniform coating in which the polylysine solution covered the entire coverslip area, were chosen and used. Experiments were performed only in the middle portion of the coverslips. In accordance with the supplier protocols, the cells (*in separate coverslips*) are incubated for 1h at room temperature with either [*conjugated Alexa Fluor 488 anti-human CD4 Ab] diluted 1:20* to characterize the expression of CD4; or [*conjugated FITC- anti-human CCR5 Ab*] diluted 1:20 to characterize the expression of CCR5. Coverslips were then rinsed 3 times for 5 min. with 1X PBS using sterilized micropipette, and were then ready to mount on slides with a drop of mounting medium and then sealed with nail polish.

In another part of the experiments, to examine the CCR5 N-terminal binding in response to an electrical stimulation, primary antibody *3A9* that recognizes epitopes in the N-terminal domain in combination with conjugated secondary antibody [*Alexa Fluor 555 IgG Ab*] was used in immunofluorescence assays. Stimulated cells and the unstimulated cells as control case were fixed with 2% PFA and treated as above were then incubated either for 3h at room temperature or in a humidity chamber environment overnight at 4°C, with both primary [*unconjugated Ab 3A9*] *diluted 1:500* and [*conjugated FITC-anti-human CCR5 Ab*] *diluted 1:20*. The coverslips were then rinsed 3 times for 5 min with 1X PBS using a sterilized micropipette. Then [*conjugated secondary antibody Alexa Fluor 555 IgG Ab*] *diluted1:1000*, was added to the sample. The coverslips were then covered by aluminum foil and incubated for 1h, at room temperature, in the dark. They were next rinsed 3 times for 5 min with 1X PBS using sterilized micropipette. The coverslips were now ready for mounting on slide with a drop of mounting medium and sealed with nail polish.

**FIGURE 9.**
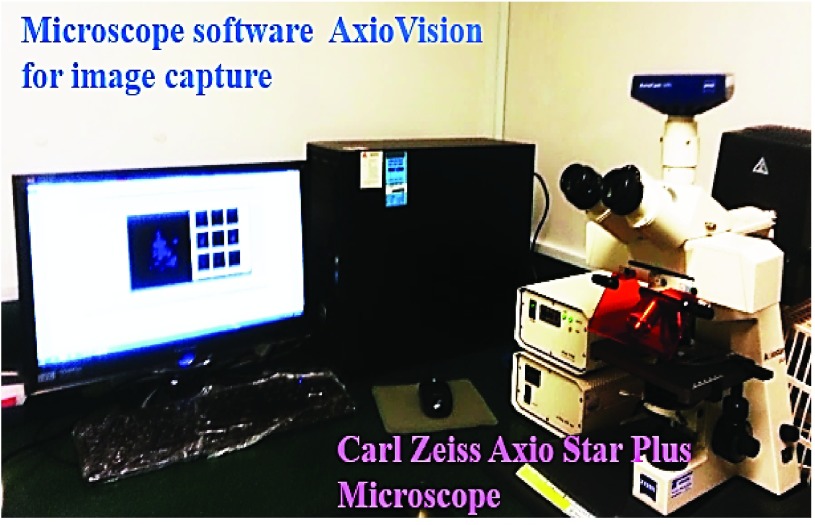
Experimental setup for acquiring images. A Carl Zeiss Axio Star plus microscope with AxioVison software.

In order to study the distribution of FGNup153 in response to electrical stimulation, stimulated cells and the unstimulated cells as control case were also examined using primary purified [*anti-Nup153 Ab*] in combination with secondary antibody [*Alexa Fluor 555 goat anti-mouse IgG*] or [*Alexa Fluor 488*] to recognize Nup153 and its distribution pattern. In accordance with the supplier protocols, cells were fixed by incubation for 15min at −20°C in cold 100% methanol, and were then rinsed 3 times for 5 min with 1X PBS using sterilized micropipette. To conform the intercellular staining, cells were also fixed by incubation for 10 min at room temperature with 4% PFA and were then rinsed 3 times for 5 min with 1X PBS using sterilized micropipettes. Then after permeabilization by incubation for 5min at room temperature in 0.5% Triton X-100 in PBS, coverslips were rinsed 3 times for 5 min with 1X PBS using sterilized micropipette. For optimum results, cells were incubated in a humidity chamber overnight at 4°C with primary purified [*anti-Nup153 anti-human Ab*] *diluted 1:250*, and then incubated in the dark with conjugated secondary antibody [*Alexa Fluor 555(red)labelled goat anti-mouse IgG*] or [*Alexa Fluora 488(green)labelled goat anti-mouse IgG*] diluted 1:1000. Coverslips were then rinsed 3 times for 5 min in 1X PBS using sterilized micropipettes. Nuclei were counterstained with DAPI (blue) solution following the BioLegend protocols by first incubating the cells with PBS for 15 minutes. The excess PBS was then removed from the coverslips and they were covered with DAPI (300 nano-moles/litre) in PBS solution and incubated for 5 minutes. Coverslips were then rinsed with PBS several times to remove all the free DAPI. Subsequently, microscope slides (S8400-1PAK) were rinsed with distilled water and were allowed to dry. All coverslips were then mounted on the clean slide with one drop of fluoroshield mounting medium, and were then sealed with nail polish. In this step, individual coverslips were mounted on parafilm and the remaining coverslips were left in 1X PBS, because the cells would dry-out which affects the results. The images were acquired for all coverslips, using a Carl Zeiss Axio Star plus microscope and analyzed using AxioVison software (as shown in Fig. 9). These sealed coverslips can be used immediately or stored for 1 year at −4°C. All the above experiments were repeated many times and several cases were examined to confirm the results through statistical analysis of the experimental data.

## V. Electrical Stimulation Test Results

In-vitro tests were conducted to examine the stimulation effect of 5Hz, 10Hz and 1MHz 2Vpp square waveform pulses on buffy coat samples. Comparison with unstimulated control samples under rigorously same experimental conditions was also carried out to investigate the effect of ELF pules on host blood cellular protein’s domain regions that could transform immune activity. Initially, leukocyte-rich buffy coat samples, and the immunofluorescence technique were utilized with fluorescent labelled protein specific antibodies to separately characterize the cell surface expression of glycoprotein CD4 and co-receptor protein CCR5. Since the anti-bodies only bind to the protein of interest, presence of other surface proteins will not effect the demonstrated expression of these two proteins. Also, the employed technique ensured that the target proteins does not undergo any denaturation. Experiments were also conducted using an appropriate binding epitope utilizing specific *mAb*, to explore CCR5 activity and the hypothesis of conformational (structural) changes due to electrostatic interactions in its predominant N-terminal protein that interacts with the V3-loop of HIV-1. In addition, the redistribution of FG-Nup153 that facilitates passage of large viral particles through the NPCs, as well as structural change in NPC was also investigated. The analysis was performed under the fluorescence microscope by isolating regions of interest and capturing images of those areas with a 100X objective.

### A. Electrical Field Intensity in the Medium

**FIGURE 10.**
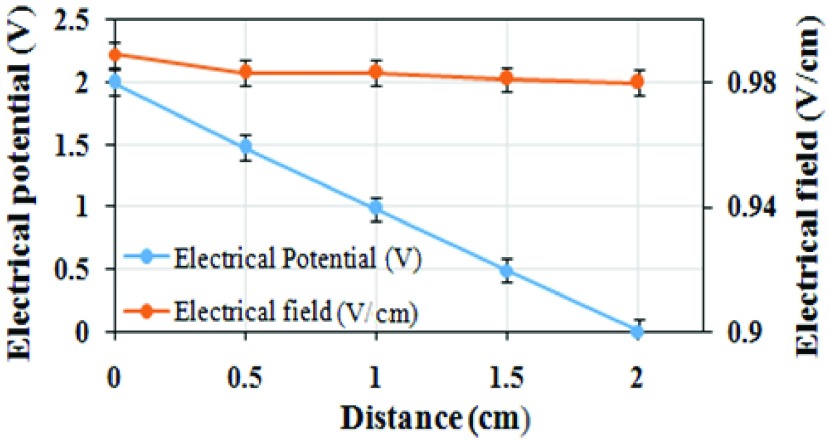
Measured electrical potential during electrical stimulation test and the corresponding electrical field strength in the medium due to the 10 Hz 2Vpp applied pulses.

Fig. 10 shows the measured electrical potential as well as the calculated field intensity in the medium from the measurement configuration in Fig. 6. The experimental measurements were carried out several times and the results were confirmed from three repeated trials. The measured values were nearly identical for the applied 2Vpp pulses at 5Hz and 10Hz. Hence, the observed electric-field strength is almost uniform and has its maximum in the range of around 1V/cm. A very small droop (around 0.009V) in the field strength is observed with separation which may be due to the influence of the surrounding medium. The possibility of heating effect and increase in temperature during the 2 hour stimulation period, due to the electric-field energy supplied to the medium has also been analyzed. Energy density ($w$) has been calculated from the measured electric field, ($E$), the pulse duration, ($\boldsymbol {\lambda }$), and the conductivity, ($\sigma$), of the medium using a formula [$w=E^{2}.\boldsymbol {\lambda }.\sigma $] in [41]. Considering, $\sigma =27.4 \times 10 ^{-6}S/m$ [28] and $w = 1.972.8 \times 10^{-3}$ *J/cm*^3^, it is evident that at low field intensity, the energy density is reasonably low. Consequently, the increase in the temperature ($\Delta T$) based on a formula [$\Delta $*T* = *w/c.* $\rho $] from [41] is around $0.538\times 10^{-3}$ *K,* which reveals that there is no significant increase in the temperature of the medium due to the low supply-voltage employed. In the above formula, *c* = *3.594 J/g.K* and $\rho =$ *1.019 g/cm*$^{-3}$ are respectively the specific heat capacity of the blood [42] and the white blood cell density [43]. The energy absorption rate due to the ELF stimulated electric field will be far lower compared to an RF generated electric field.

### B. Expression of Glycoprotein CD4 and Co-Receptor Protein CCR5

**FIGURE 11.**
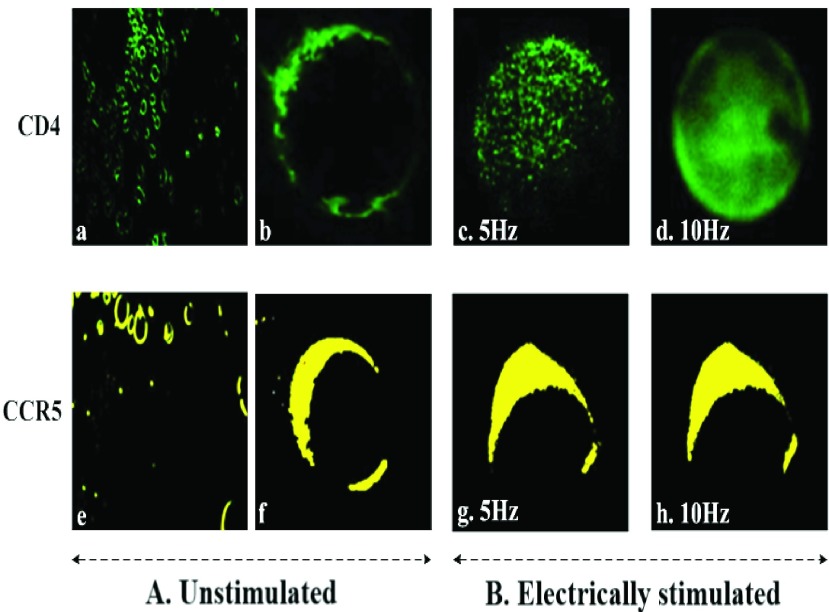
Immunofluorescence micro-photo of cell surface expression of glycoprotein CD4 (green) and co-receptor protein CCR5 (yellow) in buffy coat samples, for unstimulated (A) (“*sham* exposure” control case) and electrically stimulated (B) cells in response to ELF simulation. The intensity and distribution of cell surface fluorescence represents the concentration of CD4 and CCR5 expressions for unstimulated cells in (b) and (f), and for electrically stimulated cells in (c), (d), (g) and (h). Images (a) and (e) displays a wider view of the unstimulated CD4 and CCR5 cell populations.

**FIGURE 12.**
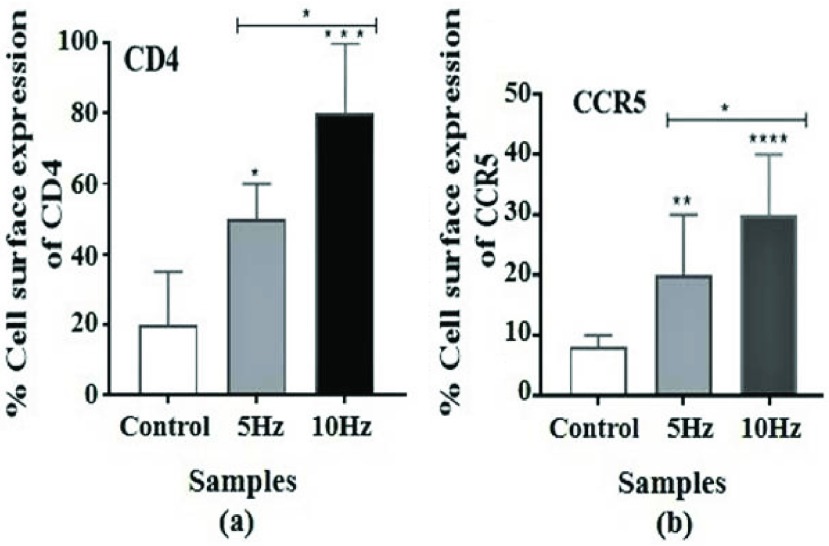
Statistical analysis of cell surface expression of (a) CD4 and (b) CCR5 for stimulated cells compared to unstimulated “*sham* exposure” controls from two separate sets of 5 independent experiments separately for CD4 and CCR5. Asterisk mark (*) at P = 0.0122 < 0.05, Asterisk mark (**) at P = 0.0049 < 0.05, Asterisk mark (***) and Asterisk mark (****) at P = 0.0001 < 0.05 represents significant difference (One way ANOVA Kruskal-Wallis Test followed by Tukey’s Multiple Comparison Test). Data are presented as median with 95% CL.

Fig. 11 shows the immunofluorescence photomicrograph of the cell surface expression of glycoprotein CD4 (green) and co-receptor protein CCR5 (yellow) for unstimulated cells (A) (without exposure to electric field) and electrically stimulated cells (B) (with exposure to electric field). Similarly labelled unstimulated samples of the same blood were used as controls under same experimental conditions (including contact to electrodes) but without application of electric field. Several regions of the individually stained coverslips were examined for each condition. The intensity and distribution of the antibody labels [*Alexa Flour 488(green)-anti-humanCD4 Ab*] for CD4 or [*FITC (yellow)-anti-human CCR5*] for CCR5 on individual cells in a population of buffy coat samples was obtained. Fig. 11(a) and (e) displays a wide view of the CD4 and CCR5 expressions of unstimulated cell populations (control case). Interestingly, the result indicates that the intensity of CD4 expression on the cell surface in response to 5Hz stimulation in Fig. 11(c) increased slightly compared to the unstimulated cell in Fig. 11(b). In Fig. 11(d) the CD4 expression intensity in response to 10Hz stimulation was much higher compared with both Fig. 11(b) and (c), and, became concentrated, regularly distributed and localized on the cell surface. The enhancement of CD4 expression due to application of ELF may be due to the change in cellular membrane potential, causing direct and instant conformational changes that affect subcellular mechanisms. In case of CCR5 test, expression of CCR5 for unstimulated cells (A) was varied on the cell surface, with some cells roughly expressing a little bit. Cell surface expression of CCR5 also varied after electrical stimulation (B), but was redistributed and clustered on the cells’ surface as shown in Fig. 11(g) and (h), compared to the unstimulated cells in Fig. 11(f). This suggests that the electrical stimulation elevated the regional density of the CCR5 more than increasing its cell surface expression, and induced redistribution of the CCR5 protein, which provides evidence that there could be a direct effect of the electrical simulation on CCR5. This may be due to the formation of clusters of some non-polar groups that are surrounded by other non-polar groups [4]. This may also be due to the fact that, the charged groups, carboxyl (COO^−^) and amino (NH_3_^+^), as well as the various side-chain charge distributions of the CCR5 protein (as discussed in section III) that interacts with the electric field might induce redistribution of the ionic bonds and realignment with the applied field, leaving the non-polar groups free and hence clustered resulting in partial alteration of the protein structure of CCR5. This result thus suits the hypothesis of possible conformational change in cellular proteins due to interactions between the charged protein on the CCR5 surface and the electric-field. For fixed cells subjected to 1MHz stimulation no staining was observed for both, anti-human CD4 and anti-human CCR5. The cell surface expression images for stimulated cells shown in Fig. 11 were confirmed and compared to unstimulated case (controls) from independent experiments by examining several regions of the individually stained coverslips. There were five independent experiments for CD4, and, separately five independent experiments for CCR5. The statistical analysis from these two sets of five independent experiments confirming the experimental outcomes is shown in Fig. 12. The Zeta ($\zeta$) potentials for CD4 and CCR5 due to the ELF stimulation were approximately calculated (using the Smoluchowski equation) to be roughly in the range of 39 mV and 86 mV respectively, the difference being mainly due to the difference in their dielectric constants. This is indicative of high cell surface charge due to the expression of these proteins. However, it also indicates that the molecular mechanisms as well as biochemical pathways for the expression of CD4 and CCR5 are possibly different.

### C. Conformational Alteration and Binding Activity in the N-Terminal Domain Epitopes of CCR5

**FIGURE 13.**
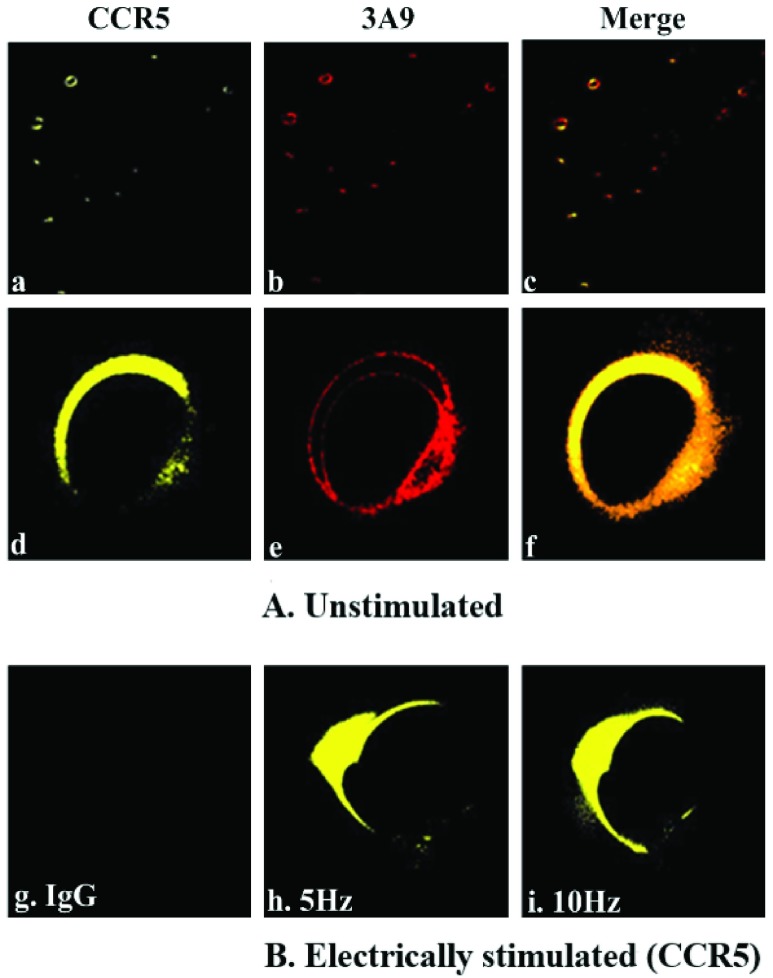
Immunofluorescence assay test for binding activity of CCR5 N-terminal domain, for unstimulated cells (A) (“*sham* exposure” control case) and electrically stimulated cells (B) in response to ELF stimulation, to investigate protein structural alterations due to electrostatic interaction. The images (a), (b) and (c) show a wider view of the unstimulated (A) cell population. The N-terminal specific binding of CCR5 (yellow) in (d) with *3A9* (red) in (e) is shown in orange (merged) in (f) for unstimulated cells (A), while, (g) represents IgG as a control, (h) and (i) represents the CCR5 (yellow) in response to electrical stimulation (B) of 5Hz and 10Hz respectively indicating no N-terminal specific binding with CCR5.

**FIGURE 14.**
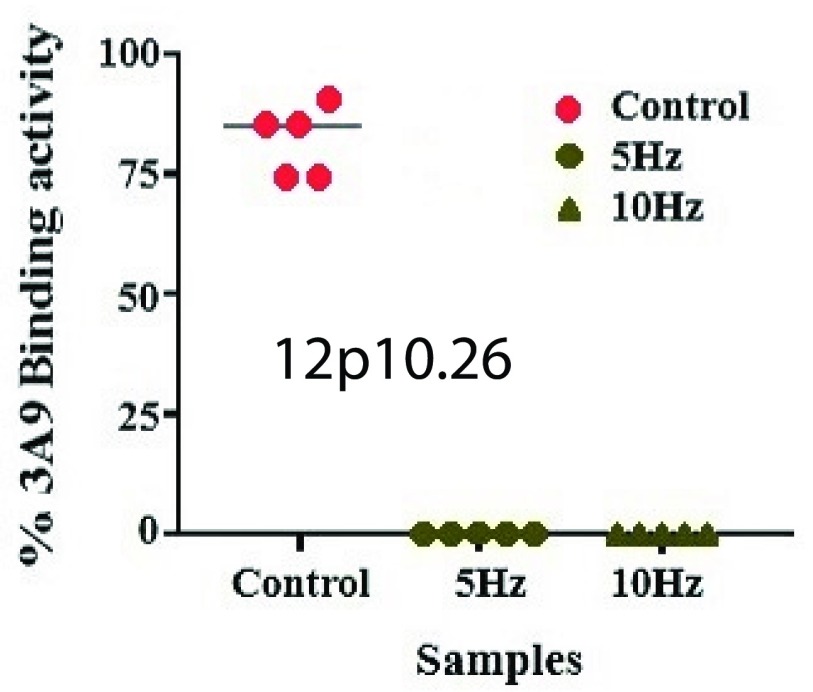
Statistical analysis of binding activity for electrically stimulated cells compared to “*sham* exposure” controls from five independent experiments. Here, $P = 0.0001< 0.05$ (One way ANOVA Kruskal-Wallis Test followed by Tukey’s Multiple Comparison Test). Horizontal lines within the plots denote median.

To examine whether ELF pulse induces redistribution of the CCR5 proteins and alters the binding site of *3A9* epitope in its N-terminal domain, leukocyte-rich buffy coat cells were assayed with *mAb 3A9* directed against the N-terminal domain of CCR5, and the binding of *3A9* epitope (binding site) in this region was explored. This *mAb* is selected because it is previously known to assay and recognize epitopes in the residues and mutate CCR5 N-terminal domains that are critical for HIV-1 viral binding activity [14]. It was also used to examine drug-bound CCR5s capability against drug-resistant viruses that uses the drug bound co-receptor for entry [44]. Electrically stimulated cells were treated according to the protocol described above and co-stained with [*FITC(yellow)*-*anti-human CCR5*] and [*mAb 3A9*], followed by secondary [*Alexa Fluor 555(red) labelled goat anti-mouse IgG*], directed against the N-terminal domain of CCR5. Similarly labelled unstimulated buffy coat samples of the same blood were used as controls for comparison under rigorously same experimental condition. Fig. 13 shows the immunofluorescence microscopy experimental results of the binding activities of *3A9* epitope (red) against the N-terminal domain of CCR5 (yellow). Many regions in the area of interest on coverslip were considered; cell surface expression of CCR5 also varied after electrical stimulation (B) and was redistributed and clustered on the cell surface compared to the unstimulated cells (A) (control case). Expressed CCR5 for cells stimulated at 5Hz displayed no surface staining with *mAb 3A9* as shown in Fig. 13(h) compared to unstimulated cells (control case in Fig. 13(f)) that were stained at the CCR5 N terminus with an orange variant of the yellow fluorescent. N-terminal specific co-localization of CCR5 (yellow) in Fig. 13(d) with *mAb 3A9* (red) in Fig. 13(e) is shown in orange (merged) in Fig. 13(f) for unstimulated cell (A) (control case). For fixed cells stimulated with 10Hz as shown in Fig. 13(i) results were similar to the 5Hz case. The result reveals that the lack of the *mAb 3A9* binding site which has the most contact residues for gp120, diminishes interactions with the CCR5 N-terminal domain polar and ionised (negatively charged) side chains, specifically Y^10^D^11^, tyrosine Y^10^, Y^14^, Y^15^ and aspartic acid D^11^ [44]. This is because *mAb 3A9* had not recognized a conformational epitope in the CCR5 N-terminal domain, and hence, [*mAb 3A9*] antibodies lost their binding ability, suggesting conformational alteration of *3A9* epitope, which may be due to the application of ELF electric field. For fixed cells stimulated with 1MHz poor staining by anti-human CCR5 resulted and hence showed no binding activities with [*mAb 3A9*]. Also, no labelling was shown in Fig. 13(g), using IgG as a control isotype-matched monoclonal antibody. A wider view of the cell population is shown in Fig. 13 (a), (b) and (c) respectively. The tests for CCR5 N-terminal binding were repeated five times independently, and, the assay test images as shown in Fig. 13 were procured five times. The statistical analysis confirming the results from these five independent experiments is shown in Fig. 14 which is quantified as a percentage of the total intensity of the cell. This result could support the hypothesis of possible structural changes in the CCR5 N-terminal domain that is involved in HIV-1 viral entry, in response to an ELF electric-field by redistribution of its protein due to electrostatic interaction. This ELF effect might, as an hypothesis prompt antiviral state for a period of time through change in the electrostatic state of the domain proteins.

### D. Distribution of FGNup153

**FIGURE 15.**
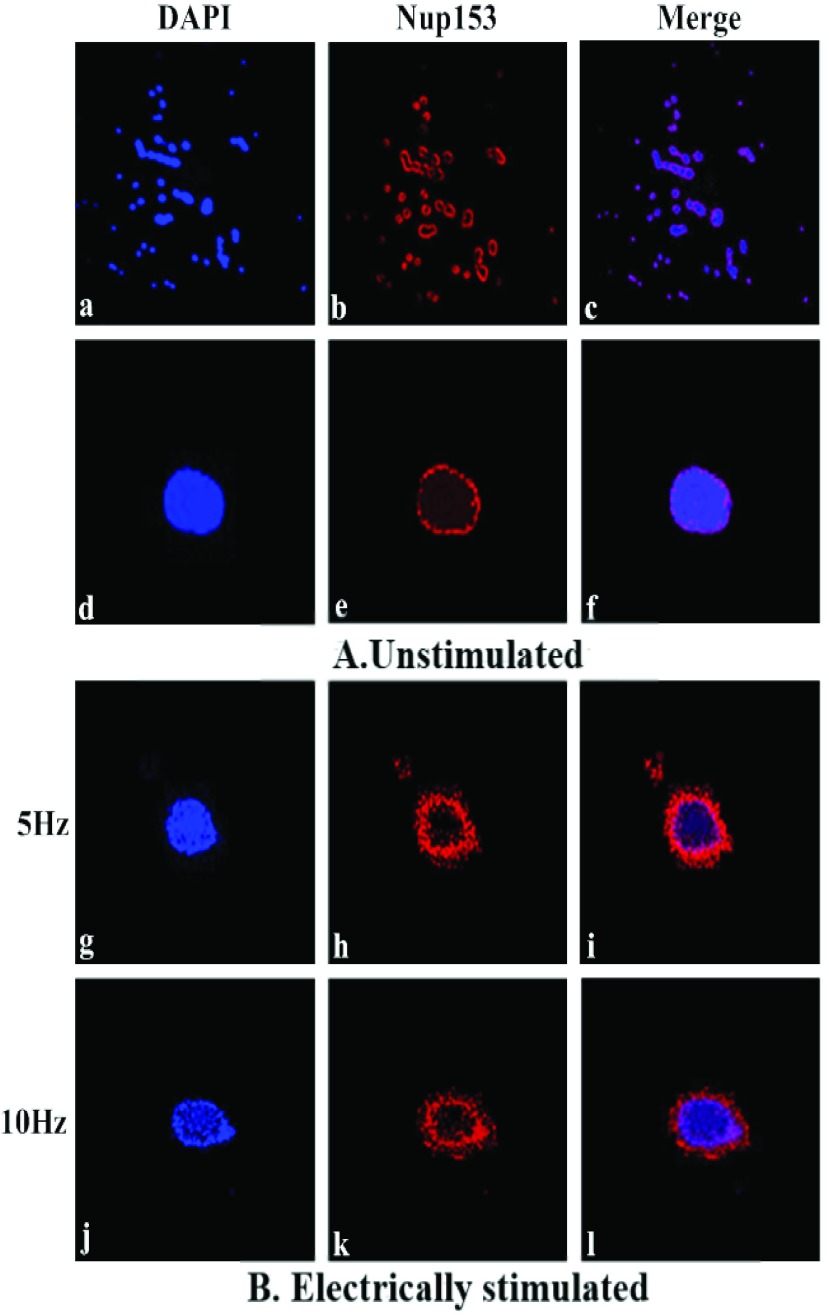
The immunofluorescence microscope images of the distribution and pattern of the FGNup153 and associated structural change due to electrostatic interactions, in response to ELF stimulation. The images of DAPI (blue) in (a) and (d) along-with Nup153 (red) in (b) and (e) is shown merged (purple) in (c) and (f), for unstimulated cells (A), where, (a), (b) and (c) is showing wider view of the cell population. The co-localization of FGNup153 with *mAb* for cells electrically stimulated (B) with 5Hz and 10Hz, are displayed in (g), (h), (i) and (j), (k), (l) respectively.

**FIGURE 16.**
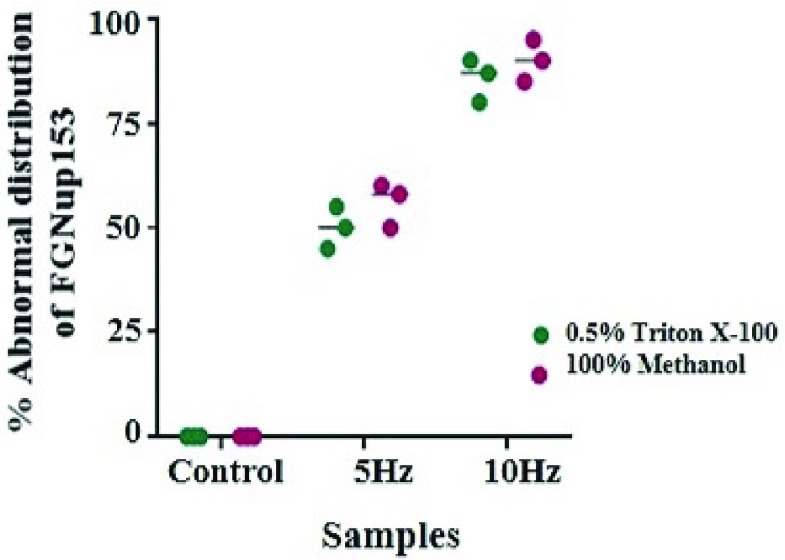
Statistical analysis of the abnormal distribution of FGNup153 for electrically stimulated cells compared to “*sham* exposure” controls from six independent experiments for the two different fixation and permeabilization approaches (0.5% Triton_X-100 and 100% Methanol). The abnormal distribution of the FGNup153 within nuclear pore membrane is by around 50% for the cells stimulated at 5Hz, and around 90% for cells stimulated at 10Hz for the two approaches. Here, $P < 0.05$ (Two way ANOVA Test followed by Bonferroni’s Multiple Comparison Test). Horizontal lines within the plots indicate the median.

As discussed earlier (section II.B), FGNup153 has dynamic roles in NPC assembly and nucleocytoplasmic traffic of large macromolecules of HIV-1 PIC and unspliced viral transcripts. Since electrostatic interactions between transporters (cargo complexes) and intrinsically disordered domains of FG-Nups are considered the main driving forces that stimulate translocation of cargos through the NPC, the dependence on translocation could be disturbed by electrostatic interactions between external ELF pulses and the FGNup153 domains resulting in structural change. This can establish an effective and temporary permeability barrier across the NPCs which could inhibit the reorganisation of NPCs and hence, the import and export of high-weight molecular HIV-1 particles. To examine this, the distribution of FGNup153 and any structural change in NPC was investigated in response to specific electrical stimulation using immunofluorescence technique with a fluorescent labelled antibody [*mAb Nup153*]. Buffy coat cells were assayed with [*mAb Nup153*] which recognises FG-repeats comprising Nup153 NPC proteins. Treated and fixed cells were electrically stimulated using 5Hz, 10Hz and 1MHz 2Vpp pulses for 2h. Stimulated cells and the unstimulated cell samples as control-case were fixed and permeabilized by two approaches (0.5% Triton_X-100 and 100% Methanol) as discussed in section IV.E. The cells were then stained with primary purified [*anti-Nup153 anti-human mAb*] followed by secondary *Ab* [*Alexa Fluor 555 (red) labelled goat anti-mouse IgG*] or [*Alexa Fluor 488 (green) labelled goat anti-mouse IgG*] as discussed earlier. Similarly labelled unstimulated buffy coat samples of the same blood specimen were used as controls for comparison under rigorously similar experimental conditions. The cells were then stained with DAPI (blue) and used as counter-stains for two different reasons; (1) to reduce the background of the fluorescence and for identification of cell-cycle, and (2) specifically staining nuclei but not cytoplasm in order to have data regarding signal localization [38]. Fig. 15 shows the immunofluorescence microscope images of the distribution and pattern of the FGNup153 that brightly highlights the nuclear membrane. The images in (a) and (d) of DAPI (blue) along-with Nup153 (red) in (b) and (e) is shown merged (purple) in (c) and (f), displaying respectively a wider view of the cell population, and of the individual cell for unstimulated case (A) (control case). The co-localization of FG-Nup153 with *mAb* for electrically stimulated cells (B) at 5Hz and 10Hz that were incubated in 100% methanol are displayed in Fig. 15(g), (h), (i) and (j), (k), (l) respectively. The results were confirmed through six independent experiments, for the two different cell fixation and permeabilization methods. There were three independent experiments for the case of cell fixation with 100% Methanol, and also, three independent experiments for the case of cell fixation with 4% PFA in PBS along-with permeabilization with 0.5% Triton_X-100 in PBS. The statistical analysis for these six experiments is shown in Fig. 16. The brightly stained antibody highlights the nuclear membrane (red) showing that the Nup153 antibody is attached and accumulates on the nuclear rim of the NPC. Electrical stimulation with 5Hz in Fig. 15(i) and 10 Hz in Fig. 15(l) affects the pattern of the FG-Nup153 by inducing structural redistribution of the FG-Nup153 and abolishing the assembly of a regular distribution around the nuclear membrane as compared to unstimulated cells (control case) in Fig. 15(f). For cells stimulated at 1MHz showed no staining for either of the two fixation and stabilization methods. The images for stimulated cell displays structurally disorganized distribution of Nup153 and considerable reassembly of its pattern, forming irregular concentrations within nuclear membrane. This suggests that the hydrophobic sequences’ association with amino-acid of Nup153 FG-repeats has collapsed, and is now irregularly and locally distributed around the nuclear membrane indicating structural change. This is because, FGNup153 is a complex containing high and low charges and polar residues with hydrophobic residues. The key role of the charged and polar sequences of the FG-repeats is to prevent the hydrophobic sequences from self-accumulating along their AA sequences and collapsing [45]. This result indicates that, electrostatic interactions between the intrinsically disordered charged and polar domains of the AAs within the FG-repeat and the ELF electric-field induce structural rearrangement, realignments and polarization of their charged and polar residue regions, in which their hydrophobic sequences become free. Hence, decreasing the ratio of charged and polar residues to hydrophobic sequences results in the destabilisation of the hydrophobic sequence within Nup153 AA sequences, causing an increase in the hydrophobicity of its disorder domain, and accordingly collapse occurs [46]. Interestingly, the results support the hypothesis of structural change in FG-Nup153 pattern due to electrostatic interaction with an applied electric field. So, this could induce structural rearrangement in the disorder of the FG-Nup153 N-terminal domain that mediates binding of HIV-1 Vpr, and also in its disordered FxFG-rich C-terminal domain that mediates binding of HIV-1 CA N-terminal domain as well as its Rev N-terminal domain. The HIV-1 Vpr and Rev domain acts like proteins that access the NPC by interacting and binding directly to FG-Nup153 domain terminals, and motivates the transport of HIV-1 particles through the NPC. Impairment of FG-Nup153 could disturb the reorganisation of NPCs and the factors associated with the passage through NPCs. On the other hand, since the positively charged region of the FG-Nups disordered domains interacts with the negatively charged cargo complex (translocating particles) to facilitate their passage through the NPC, the electric field that aligns with their polar and charged regions could possibly induce structural redistribution in the other FGNup networks of NPC thus preventing the HIV-1 from using other NPC entry mechanisms. Hence, the import and export functions becomes inefficient for a limited period of time, with the applications of an ELF electric field which could destroy the life-cycle replication of HIV-1 virus.

## VI. Discussion and Conclusion

In this work, the underlying domain region protein-protein electrostatic interaction mechanisms of the HIV-1 virus and the host cell was considered. A novel concept of low-voltage ELF stimulation of human blood cellular proteins has been developed to investigate the hypothesis of conformational change owing to the conductivity and permittivity properties of these proteins, leading to transformation of immune activity. The mechanistic understanding of ELF field effect on CCR5 as well as nucleocytoplasmic transport is limited by the lack of experimental methods to directly monitor conformational change and dynamics of CCR5 and FG-Nup153 in-situ during the electrical stimulation, as well as the complexity of CCR5 and NPC. Thus, use of the immunofluorescence assay technique in this work to support the hypothesis of possible conformational change is significantly applicable. The results motivate possible *clinical application* of ELF electrical stimulation for enhancing immune activity (e.g., through increased cell surface CD4 expression) or possibly inhibiting virus and host-cell interaction, which could be employed in-vivo as an anti-HIV-1 treatment. There are two translational aspects that could possibly encourage electro-medical stimulation treatment, (1) stimulating the natural healing process, and, (2) inducing change in the charge distribution of biologically active molecules, thus disturbing the virus-host cell interaction, and hence, the virus life cycle, possibly leading to inactivation signal induced virus death. Based on the above investigation of the disease mechanisms and its solution from an electrical point of view, is a new contribution, and may possibly provide an alternative treatment. Although further investigations are required in order to use the technique of ELF electrical stimulation for in-vivo treatment, the presented results are promising enough and provides the necessary impetus for consideration of ELF electrical stimulation as an enabling technique for human immune response.

## Appendix

see Fig. 8–16.
